# A Real-Time Interference Monitoring Technique for GNSS Based on a Twin Support Vector Machine Method

**DOI:** 10.3390/s16030329

**Published:** 2016-03-04

**Authors:** Wutao Li, Zhigang Huang, Rongling Lang, Honglei Qin, Kai Zhou, Yongbin Cao

**Affiliations:** 1 School of Electronics and Information Engineering, Beihang University, Beijing 100191, China; lwt_beihang@buaa.edu.cn (W.L.); baahzg@163.com (Z.H.); ronglinglang@163.com (R.L.); 2 Academy of Opto-Electronics, Chinese Academy of Sciences, Beijing 100094, China; zhoukai@aoe.ac.cn; 3 Realsil Microelectronics Inc., Suzhou 215021, China; cao121917@126.com

**Keywords:** global navigation satellite system, interference monitoring, twin support vector machine

## Abstract

Interferences can severely degrade the performance of Global Navigation Satellite System (GNSS) receivers. As the first step of GNSS any anti-interference measures, interference monitoring for GNSS is extremely essential and necessary. Since interference monitoring can be considered as a classification problem, a real-time interference monitoring technique based on Twin Support Vector Machine (TWSVM) is proposed in this paper. A TWSVM model is established, and TWSVM is solved by the Least Squares Twin Support Vector Machine (LSTWSVM) algorithm. The interference monitoring indicators are analyzed to extract features from the interfered GNSS signals. The experimental results show that the chosen observations can be used as the interference monitoring indicators. The interference monitoring performance of the proposed method is verified by using GPS L1 C/A code signal and being compared with that of standard SVM. The experimental results indicate that the TWSVM-based interference monitoring is much faster than the conventional SVM. Furthermore, the training time of TWSVM is on millisecond (ms) level and the monitoring time is on microsecond (μs) level, which make the proposed approach usable in practical interference monitoring applications.

## 1. Introduction

The Global Navigation Satellite System (GNSS), which is a crucial means for navigation and positioning for both civil and military applications, is playing an increasingly significant role in modern society. However, the GNSS signals are extremely fragile and vulnerable, and can easily be influenced by interferences [1]. Therefore, interference monitoring techniques are exceedingly essential for improving the anti-interference performance of GNSS receivers. GNSS interference monitoring must be accurate and real-time. There are plenty of common methods for interference monitoring, such as the hypothesis testing method, method based on characteristic parameter threshold, integrated navigation method, and smart antenna technology.

### 1.1. Hypothesis Testing Method for Interference Monitoring

The hypothesis testing method establishes hypothesis testing statistics with monitoring indicators, and obtains the threshold of the monitoring indicators with the given significance level. In [2], Balaei and Dempster proposed a statistical inference technique for GPS interference detection, where the statistical parameters of the signal without interference were obtained and then the presence of interference was tested by comparing these parameters as a null hypothesis with the parameters extracted from a later window of data. The indicators utilized to establish hypothesis testing statistics include power spectral density, the correlator output, code phase, *etc.* [2,3]. The hypothesis testing method requires the predictable interference characteristics when making a hypothesis in order to ensure the rationality of the corresponding statistics.

### 1.2. Interference Monitoring Method Based on Characteristic Parameter Threshold

The method based on characteristic parameter threshold compares the actually measured indicators or monitoring quantities with a preset threshold in order to determine whether there are interferences. The monitoring indicators include the quantization weight gain of the A/D converter [4], power spectral, AGC (automatic gain control) [5] carrier to noise ratio [6], the correlator output [7], *etc.* This method requires a monitoring threshold, which is usually derived from the corresponding characteristic of the GNSS signals without interference. However, the GNSS signal environment is always complex, and especially during transfer, the GNSS signal inevitably suffers from intentional or unintentional interferences. Therefore, utilizing the characteristic without interference as the threshold will increase the false alarm rate and missing report rate.

### 1.3. Integrated Navigation Method for Interference Monitoring

In 2011 Faurie and Giremus proposed an interference monitoring method based on GNSS and Inertial Navigation System (INS) [8], where GNSS is usually integrated with INS using an extended Kalman filter. The least squares estimated values of the potential variance jump are calculated by studying the impact on the covariance of the Kalman filter outputs induced by the increase of the GNSS noise. With these estimated values as the inputs of continuous Bayesian testing experiments, the presence of interference in the received GNSS signals can be determined according to the experimental results. This method requires the GNSS and INS to work collaboratively, and superior stability of the system as well. Nevertheless, the existence of interferences will inevitably affect the stability of receivers.

### 1.4. Smart Antenna Technology for Interference Monitoring

Wilson and Ganguly proposed in 2006 a GPS interference monitoring method based on 3D Controlled Reception Pattern Antennas (CRPA) [9], where an implementation of Space-Time Adaptive Processing (STAP) algorithm using FFT was presented. The experimental results indicated that this algorithm could achieve a preferable monitoring effect under lower interference power conditions.

Among all these methods, the hypothesis testing method and the method based on characteristic parameter thresholds require theoretical values or models without interferences. However, due to the complicated environment, the time-varying and uncertain GNSS signal can make it difficult to precisely establish models and determine the theoretical values. As a result, we cannot just rely on the traditional methods to establish the necessary precise models to monitor the interferences. In addition, the integrated navigation method and smart antenna technology require superior stability of systems or superior parameter deployment.

On the other hand, applying the GNSS signals will accumulate huge amounts of data, which contain the influences of interferences on the signal quality and receiver performance. We can monitor the interferences without the precise physical models if the information and knowledge can be mined from these data. This method based on GNSS signals, seeks out regularities from these data, and then utilizes these regularities to monitor the unknown or unobservable data, without any need to know the precise analytic models of the systems in advance, thus making this method a more intelligent and flexible one. At present, the data-driven method has achieved successful applications in fields such as fault diagnosis and fault prediction. In this paper, the data-driven method is introduced into the field of interference monitoring, transforming interference monitoring problems into artificial intelligence classification problems, then used to monitor the interferences.

This paper presents an interference monitoring method, where the parameters manifesting the characteristics of interferences, such as the power spectrum, carrier to noise ratio, the correlator output, *etc.*, are extracted from the GNSS signals, and then are imported into an artificial intelligence classifier to determine whether there are interferences, and to acquire the information such as the type and strength of the interferences.

The selection of the classifier has a crucial impact on the accuracy and efficiency of monitoring. Support Vector Machine (SVM) is chosen as the classifier in this paper. SVM is one of the state-of-the-art classification techniques. Benchmarking studies reveal that the SVM performs the best among current classification techniques [10].

Solving of SVM involves quadratic programming, and the computational complexity rapidly increases as the training samples increase in number. In this paper, the Twin Support Vector Machine (TWSVM) technique is utilized to ensure the interference monitoring is real-time. TWSVM has outstanding classification precision, faster than SVM, and it satisfies the accuracy requirement of the GNSS interference monitoring. Therefore, this paper will focus on the GNSS interference monitoring with the TWSVM technique.

The rest of this paper is organized as follows: in the following section an introduction to SVM is presented. The model of TWSVM and the LSTWSVM algorithm are presented in Section 3. In Section 4, the features used for the interference monitoring are introduced. In Section 5, the algorithm is tested and verified with GPS L1 band C/A signals. Finally, the conclusions are made in Section 6.

## 2. Support Vector Machine

We assume that the training sample set of the interference monitoring is $T=\{(x_{1},y_{1}),...,(x_{l},y_{l})\}\in {\left(\chi \times \gamma \right)}^{l}$, where $\left(x_{i},y_{i}\right)$, i=1,...,l are the n-dimension vectors constituted by the characteristic parameters of the interference monitoring, in which $x_{i}\in \chi =R^{n}$, and $y_{i}\in \gamma =(1,-1)$. It is defined that, when $y_{i}=1$, $x_{i}$ is the characteristic parameter with interferences and when $y_{i}=-1$, correspondingly $x_{i}$ is the characteristic parameter without interferences.

The SVM aims to build a classification hyperplane wx+b=0, making $x_{i}w\geq 1-b$ for the sample of $y_{i}=1$, and $x_{i}w\leq -1-b$ for the sample of $y_{i}=-1$. Therefore, the interference monitoring problem is transformed into the optimization problem by SVM [11] as follows:

(1) $$\begin{array}{l} \underset{w,b,\xi }{\min}\;\frac{1}{2}{\left\|w\right\|}^{2}+C\sum_{i=1}^{l}\xi_{i}\\ s.t.\;y_{i}(wx_{i}+b)\geq 1-\xi_{i},\xi_{i}\geq 0\;,i=1,2...,l \end{array}$$

where $\xi =(\xi_{1},\xi_{2}\cdots \xi_{n})$ is the relaxation factor and C is the penalty parameter. The Wolfe dual of Equation (1) is:

(2) $$\begin{array}{l} \min\;\frac{1}{2}\sum_{i=1}^{l}\sum_{j=1}^{l}\alpha_{i}\alpha_{j}y_{i}y_{j}K\left(x_{i},x_{j}\right)-\sum_{i=1}^{l}\alpha_{i}\\ s.t.\;0\leq \alpha_{i}\leq C,\sum_{i=1}^{l}\alpha_{i}y_{i}=0 \end{array}$$

where each $a_{i}$ is a Lagrange multiplier, which corresponds to the sample $\left(x_{i},y_{i}\right)$; $K(x_{i},x_{j})$ is Gaussian kernel function, which is defined as:

(3) $$K(x_{i},x_{j})=exp(-\frac{{\left\|x_{i}-x_{j}\right\|}^{2}}{\sigma^{2}})$$

Let $a_{1},\cdots ,a_{l}$ is the optimum solution of Equation (2), the classification criterion of GNSS signals to be monitored is:

(4) $$f\left(x\right)=\mathrm{sgn}\left\{\sum_{i=1}^{l}\alpha_{i}y_{i}K\left(x_{i},x\right)+b\right\}$$

As is shown, the classifier decided by Equation (4) only relies on the samples $\left(x_{i},y_{i}\right)$ when $a_{i}\neq 0$, and their input $x_{i}$ is called the Support Vector. The b in Equation (4) can be solved by:

(5) $$b=y_{j}-\sum_{i=1}^{l}y_{i}\alpha_{i}K(x_{i}x_{j})$$

where the $a_{j}$ corresponding to $y_{j}$ should meet the condition of $0\leq \alpha_{j}\leq C$.

The common method to solve Equation (2) is the Sequential Minimal Optimization (SMO) algorithm [12]. However, the training time will rapidly increase as the number of training samples increases, making it hard for interference monitoring to be in real-time. Therefore, in this paper the TWSVM technique is adopted to improve the training speed.

## 3. Twin Support Vector Machine

### 3.1. The Model of TWSVM

Unlike the standard SVM, TWSVM constructs two hyperplanes $xw_{1}+b_{1}=0$ and $xw_{2}+b_{2}=0$, and then makes the sample points with interferences as close as possible to the hyperplane $xw_{1}+b_{1}=0$, and as far as possible from the hyperplane $xw_{2}+b_{2}=0$. Likewise, the sample points without interferences are made as close as possible to the hyperplane $xw_{2}+b_{2}=0$ and as far as possible from the hyperplane $xw_{1}+b_{1}=0$.

Supposing that a n-dimension sample set $T=\{(x_{1},y_{1}),...,(x_{l},y_{l})\}\in {\left(\chi \times \gamma \right)}^{l}$ with $m_{1}$ training points with interferences, that is, +1 class of sample, and $m_{2}$ training points without interferences, that is, −1 class of sample, the +1 class of training points are denoted by the matrix $A\in {ℜ}^{m_{1}\times n}$, and the −1 class of training points are denoted by the matrix $B\in {ℜ}^{m_{2}\times n}$. The corresponding optimization problems of TWSVM are:

(6) $$\begin{array}{l} \underset{w_{1},b_{1}}{\min}\;\frac{1}{2}{\left(Aw_{1}+e_{1}b_{1}\right)}^{T}(Aw_{1}+e_{1}b_{1})+c_{1}e_{2}^{T}\xi \\ s.t\;-(Bw_{1}+e_{2}b_{1})+\xi \geq e_{2},\;\xi \geq 0 \end{array}$$

(7) $$\begin{array}{l} \underset{w_{2},b_{2}}{\min}\;\frac{1}{2}{\left(Bw_{2}+e_{2}b_{2}\right)}^{T}(Bw_{2}+e_{2}b_{2})+c_{2}e_{1}^{T}\xi \\ s.t\;(Aw_{2}+e_{1}b_{2})+\xi \geq e_{1},\;\xi \geq 0 \end{array}$$

where $c_{1},c_{2}>0$ are the penalty parameters which can be selected by the heuristic algorithm proposed in [13], and $e_{1},e_{2}$ are the column vectors with all of elements being one.

The computational complexity of the standard SVM is $Ο(n^{3})$, and each complexity of the two optimization problems Equations (6) and (7) solved by TWSVM is nearly Ο(n/2), therefore, the computational speed of TWSVM is about four times faster than that of the standard SVM.

### 3.2. The Solution of TWSVM

Currently, there are three methods to solve the TWSVM: the Sequential Minimal Optimization (SMO) algorithm [12], the Successive over Relaxation (SOR) iterative method [14,15] and the Least Squares Twin Support Vector Machine (LSTWSVM) algorithm [16]. The LSTWSVM algorithm is the fastest one, as verified by the experimental analysis, so it is utilized as the solution of TWSVM in this paper.

Based on the fact that the Support Vectors are almost distributed around the classification hyperplanes, LSTWSVM narrows the constraint of the optimization problem near to the classification hyperplane, and modifies the original optimization models Equations (6) and (7) to Equations (8) and (9), respectively.

(8) $$\begin{array}{l} \underset{w_{1},b_{1}}{\min}\;\frac{1}{2}{\left(Aw_{1}+e_{1}b_{1}\right)}^{T}(Aw_{1}+e_{1}b_{1})+\frac{c_{1}}{2}\xi^{T}\xi \\ s.t\;-(Bw_{1}+e_{2}b_{1})+\xi =e_{2} \end{array}$$

(9) $$\begin{array}{l} \underset{w_{2},b_{2}}{\min}\;\frac{1}{2}{\left(Bw_{2}+e_{2}b_{2}\right)}^{T}(Bw_{2}+e_{2}b_{2})+c_{2}e_{1}^{T}\xi \\ s.t\;(Aw_{2}+e_{1}b_{2})+\xi =e_{1},\;\xi \geq 0 \end{array}$$

Because of the relaxation factor ξ, the area confirmed by the constraints of Equations (8) and (9) is a rectangle.

Substituting the constraint into the objective function, Equation (8) becomes:

(10) $$\underset{w_{1},b_{1}}{\min}\;\frac{1}{2}{\left\|Aw_{1}+e_{1}b_{1}\right\|}^{2}+\frac{c_{1}}{2}{\left\|Bw_{1}+e_{2}b_{1}+e_{2}\right\|}^{2}$$

Differentiating Equation (10) for $w_{1}$ and $b_{1}$, respectively, and making the derivatives zero, we can obtain:

(11) $$A^{T}(Aw_{1}+e_{1}b_{1})+c_{1}B^{T}(Bw_{1}+e_{2}b_{1}+e_{2})=0$$

(12) $$e_{1}^{T}(Aw_{1}+e_{1}b_{1})+c_{1}e_{2}^{T}(Bw_{1}+e_{2}b_{1}+e_{2})=0$$

Combining Equation (11) with Equation (12), and adapting it into matrix form, then we have:

(13) $$\left[\begin{array}{cc} B^{T}B & B^{T}e_{2}\\ e_{2}B & m_{2} \end{array}\right]\left[\begin{array}{c} w_{1}\\ b_{1} \end{array}\right]+\frac{1}{c_{1}}\left[\begin{array}{cc} A^{T}A & A^{T}e_{1}\\ e^{T}A & m_{1} \end{array}\right]+\left[\begin{array}{c} B^{T}e_{2}\\ m_{2} \end{array}\right]=0$$

(14) $$\left[\begin{array}{c} w_{1}\\ b_{1} \end{array}\right]={\left[\begin{array}{cc} B^{T}B+\frac{1}{c_{1}}A^{T}A & B^{T}e_{2}+\frac{1}{c_{1}}A^{T}e_{1}\\ e_{2}^{T}B+\frac{1}{c_{1}}e_{1}^{T}A & m_{2}+\frac{1}{c_{1}}m_{1} \end{array}\right]}^{-1}\left[\begin{array}{c} -B^{T}e_{2}\\ -m_{2} \end{array}\right]$$

(15) $$\left[\begin{array}{c} w_{1}\\ b_{1} \end{array}\right]={\left[\begin{array}{l} \left[\begin{array}{c} B^{T}\\ e_{2}^{T} \end{array}\right]\left[\begin{array}{cc} B^{T} & e_{2}^{T} \end{array}\right]+\\ \frac{1}{c_{1}}\left[\begin{array}{c} A^{T}\\ e_{1}^{T} \end{array}\right]\left[\begin{array}{cc} A^{T} & e_{1}^{T} \end{array}\right] \end{array}\right]}^{-1}\left[\begin{array}{c} -B^{T}e_{2}\\ -m_{2} \end{array}\right]$$

Defining $E=\left[\begin{array}{cc} A & e_{1} \end{array}\right]$ and $F=\left[\begin{array}{cc} B & e_{2} \end{array}\right]$, Equation (15) becomes:

(16) $$\left[\begin{array}{c} w_{1}\\ b_{1} \end{array}\right]=-{\left(F^{T}F+\frac{1}{c_{1}}E^{T}E\right)}^{-1}F^{T}e_{2}$$

Similarly:

(17) $$\left[\begin{array}{c} w_{2}\\ b_{2} \end{array}\right]={\left(E^{T}E+\frac{1}{c_{2}}F^{T}F\right)}^{-1}E^{T}e_{1}$$

## 4. Interference Monitoring

**Figure 1:**
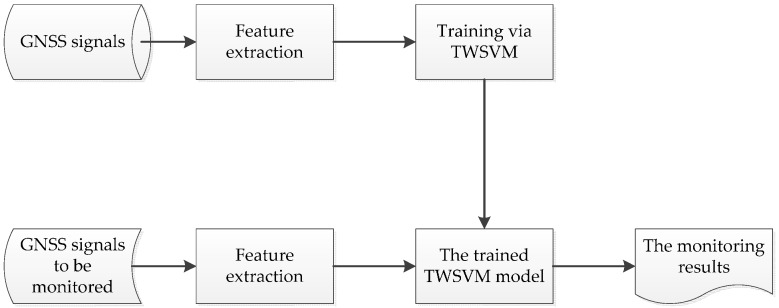
The process chart of the TWSVM-based interference monitoring.

The process of the interference monitoring based on TWSVM is shown in Figure 1. Firstly, the training samples are extracted from GNSS signals and then imported into the TWSVM to get TWSVM model; secondly the corresponding characteristic parameters are extracted from GNSS signals to be monitored, and then imported into the trained TWSVM model to get the monitoring results.

From Figure 1, we can see that it is necessary to extract the features for interference monitoring. Therefore, we firstly study feature extraction from GNSS signals. According to the position of interference monitoring indicators relative to the correlator, the monitoring indicators are divided into pre-correlation interference monitoring indicators and post-correlation interference monitoring indicators. The pre-correlation interference monitoring is mainly implemented through the extraction of the observations of receivers, including antenna, AGC gain, ADC, *etc.* The post-correlation interference monitoring is implemented by observing the carrier to noise ratio, correlator output, *etc*.

### 4.1. Power Spectral Density

Frequency characteristic is the most direct reflection of signal characteristics. Through observing the frequency characteristic of GNSS signals, a variety of information can be obtained, including signal bandwidth, power spectral density, in-band stray, and interference performance. Therefore, the analysis of frequency characteristic is firstly carried out in interference monitoring. Power Spectral Density (PSD) reflects the signal power distribution within the signal bandwidth. With the measurement of the PSD, whether the power of every frequency is well-distributed or there is abnormal power can be seen. PSD is also an effective means to monitor the GNSS signal interference.

### 4.2. Carrier to Noise Ratio

Carrier to noise ratio ($C/N_{0}$) is the ratio of received signal power to noise power spectral density. Carrier to noise ratio affects the performance such as the acquisition, tracking and data demodulation of receivers, which will ultimately affect the positioning precision of receivers. When the inference exists, it superimposes noise on the GNSS signals, thus reducing the carrier to noise ratio of receivers and increasing the measurement errors of pseudo range and pseudo range rate. This will result in the increase of the error of navigation positioning. When the interference power is too high, the carrier to noise ratio will fall below the tracking threshold, making receivers lose their capabilities of obtaining the navigation information from satellite signals. Therefore, carrier to noise ratio can be used as an interference monitoring indicator.

When inferences exist, the carrier to noise ratio is given by:

(18) $${\left(\frac{C_{s}}{N_{0}}\right)}_{eff}=\frac{\frac{C_{s}}{N_{0}}\int_{-\beta_{r}/2}^{\beta_{r}/2}G_{s}(f)df}{\int_{-\beta_{r}/2}^{\beta_{r}/2}G_{s}(f)df+\frac{C_{I}}{N_{0}}\int_{-\beta_{r}/2}^{\beta_{r}/2}G_{I}(f)G_{s}(f)df}$$

where $\beta_{r}$ is the bandwidth of receiver front-end and processing filter, $N_{0}$ is the noise PSD, $C_{s}$ is the carrier power of navigation signals, $G_{s}(f)$ is the normalized PSD of navigation signals, $C_{I}$ is carrier power of the interference signals, and $G_{I}(f)$ is the normalized PSD of interference signals.

### 4.3. Correlator Output

When no interferences exist, the correlator output is basically constant, which is decided by pseudo-code autocorrelation, correlation length of the correlator and integral time. However, interferences can make the correlator output vary. Therefore, the correlator output can be also used as an interference monitoring indicator.

#### 4.3.1. Correlator Output Power

Correlator Output Power (COP) is defined as the result of the correlator output power when the former and later correlator are balanced divided by the expected thermal noise threshold, which is given by:

(19) $$COP=\frac{I^{2}+Q^{2}}{N}$$

where I and Q are in-phase and quadrature signal of the correlator output, respectively. N is the expected noise level, varying from different receivers, which is a statistical expected value. Usually, the correlator output signal is:

(20) $$\begin{array}{l} I=\frac{A}{\sqrt{2}}C_{w}C_{rw}X_{w}\cos(\phi_{w}-\phi_{rw})\\ Q=\frac{A}{\sqrt{2}}C_{w}C_{rw}X_{w}\sin(\phi_{w}-\phi_{rw}) \end{array}$$

where, A is the amplitude of input signal, $C_{w}$ is the C/A code in the sampling rate of w, $C_{rw}$ is the local pseudo-code, $X_{w}$ is the data code in the sampling rate of w, $\phi_{w}$ is the sampling signal phase, and $\phi_{rw}$ is the local pseudo-code phase.

When there are interferences through receivers, distortion, delay or peak reduction will arise in the receiver correlation peak curve, which directly results in COP variation. Therefore, the quality of the received signals can be judged by COP and the preliminary analysis can determine whether there are interferences.

#### 4.3.2. Correlator Output Power Standard Deviation

The standard deviation of correlator output power (COPσ) can be also utilized to analyze the extent of impact on navigation signals from interferences. The COPσ is the measures of dispersion of COP, which is defined by:

(21) $$\begin{array}{l} COP\sigma =\sqrt{E{\left[COP-E(COP)\right]}^{2}}\\ \;=\sqrt{E(COP^{2})-{\left[E(COP)\right]}^{2}} \end{array}$$

When the input signals are constant, the peak value of *COP* is relatively stable. Thus the COPσ is relatively small. However, when the unstable interferences exist, the correlation peak jitters increase and the value of COPσ gets larger.

## 5. Experiments

### 5.1. Experimental Analyses of Monitoring Indicators

**Figure 2:**
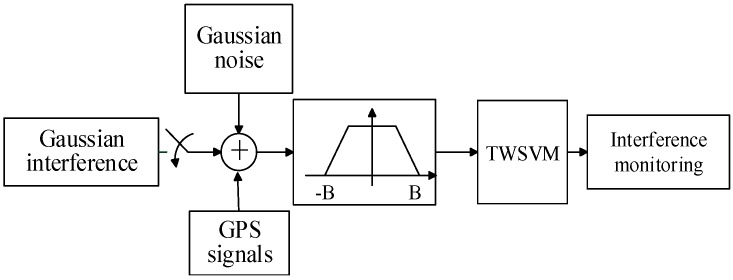
Experimental scheme of the interference monitoring.

In this section, the GPS L1 C/A code signal is tested, and the experimental scheme is shown in Figure 2. The GPS signals used in the experiment are generated by simulation, and the power ratio between GPS signals and the additional white Gaussian noise is −18 dB. Broadband Gaussian interference covers the whole frequency range of GPS signals, and narrowband Gaussian interference only covers the frequency range with the center frequency of 3.563 MHz and the bandwidth of 80 KHz. The receiver bandwidth is 2 MHz.

#### 5.1.1. Influence of Interference on $C/N_{0}$

**Figure 3:**
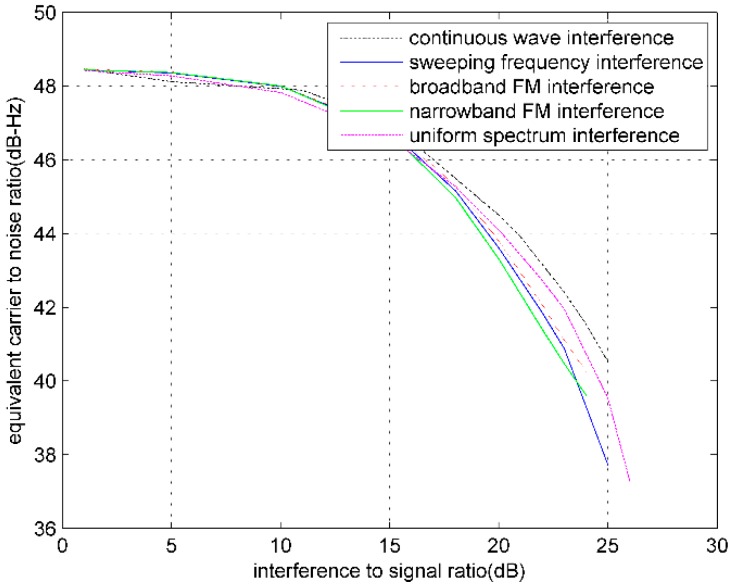
Influences of different RFIs on equivalent $C/N_{0}$.

When noise and RFIs are superimposed on the GPS digital intermediate frequency signal, with different RFIs and the increase of interference signal power, the output $C/N_{0}$ of GPS software receiver will change. Figure 3 shows the curves that express the relation between the equivalent $C/N_{0}$ and the interference to signal ratio (ISR), respectively, under conditions of continuous wave (CW) interference, broadband FM interference, narrowband FM interference, sweeping frequency CW interference, and uniform spectrum interference for the PRN3 satellite.

The experiment demonstrates that the influences on the equivalent $C/N_{0}$ from different RFIs are different, which further proves that $C/N_{0}$ can be used as the interference monitoring indicator.

#### 5.1.2. Correlator Output

**Figure 4:**
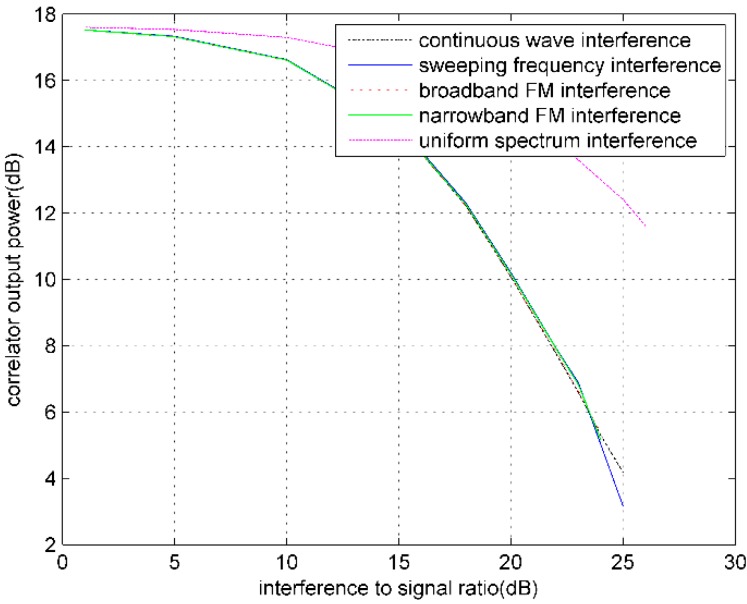
Influences of different RFIs on COP.

**Figure 5:**
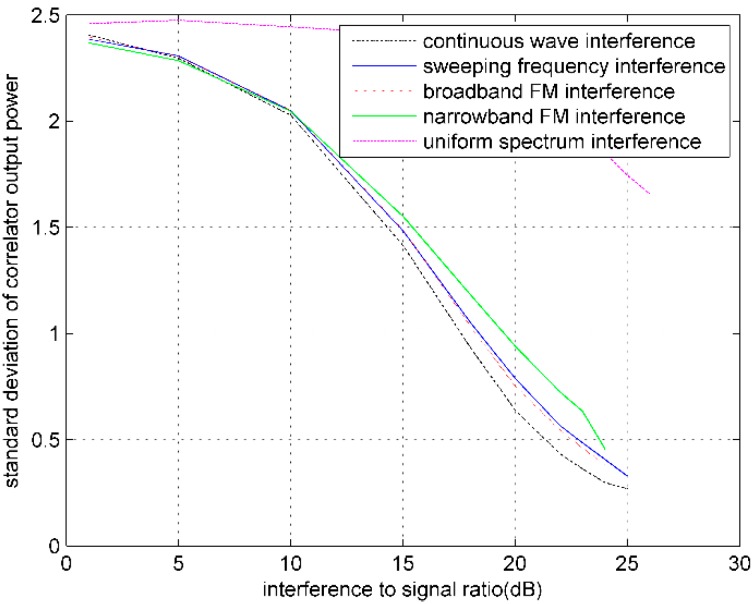
Influences of different RFIs on COPσ.

When noise and RFIs are superimposed on GPS digital intermediate frequency signal, with different RFIs and the increase of interference signal power, the output COP and COPσ of GPS software receiver will change. Figure 4 and Figure 5 show the PRN3 satellite curves of the relation between both COP and COPσ and the ISR, respectively, under conditions of CW interference, broadband FM interference, narrowband FM interference, sweeping frequency CW interference, and uniform spectrum interference.

**Figure 6:**
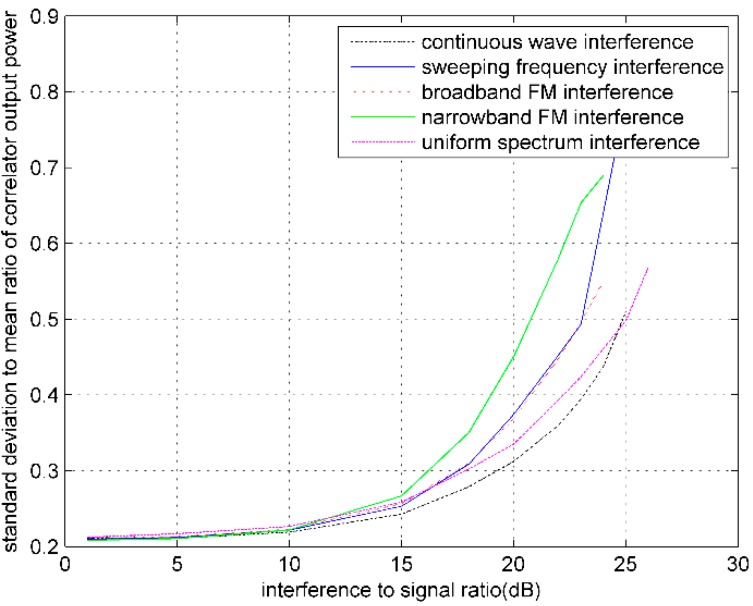
The variation curves of COPσ to COP mean ratio under different RFIs.

In order to better analyze the impact on COP from RFIs, Figure 6 depicts the relation curves of COPσ to COP mean ratio under conditions of different RFIs and interference strength.

The experimental results show that, with the increase of interference strength, COP gradually decreases until the receiver loses lock. Therefore, the COP can be used as an indicator of interference monitoring. In the experiment, the COPσ measured after the receiver stably tracking does not increase with the rising of interference strength, but show a decreasing trend This is because of the gradual decreasing of COP. As is shown in Figure 6, with the increase of interference strength, the COP mean decreases faster than the COPσ. This indirectly illustrates that the COP jitter is aggravating.

### 5.2. Experiment of Interference Monitoring

**Figure 7:**
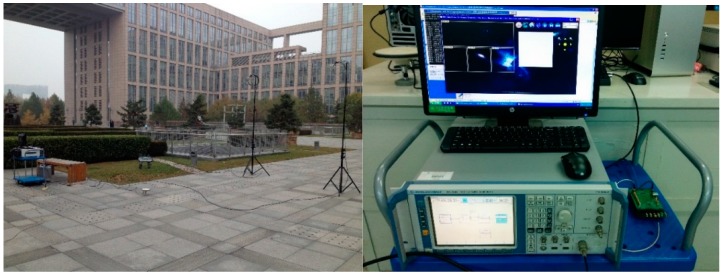
The hardware setup for the experiments.

To demonstrate the results discussed in the previous sections, three experiments were designed. The hardware setup for the experiments is shown in Figure 7. As can be seen the software receiver is used to capture the IF data to be analyzed and a SMU200A Vector Signal Generator (Rohde & Schwarz GmbH & Co. KG, Munich, Germany) is used to generate the broadband and narrowband interferences. The interferences are transmitted by a G5Ant-52PT1 transmitting antenna (Antcom corporation, Torrance, CA, USA) which are combined with the real GPS signal received by a HX-BS781A antenna (Harxon corporation, Shenzhen, China).

#### 5.2.1. Pre-Correlation

The power spectral density is utilized as the pre-correlation indicator. With the broadband and narrowband interference monitoring tests, the monitoring effect of the SVM is verified.

The testing parameters are set as follows: 

Standard SVM: penalty parameter C=10, kernel function parameter σ=1;

LSTWSVM: penalty parameter $c_{1}=c_{2}=10$, kernel function parameter σ=1.

The monitoring results are shown in Table 1 and Table 2, respectively.

Table 1 and Table 2 show the comparison of classification accuracy and speed between LSTWSVM and the standard SVM for the broadband and narrowband interference monitoring. The results indicate that both the LSTWSVM and the standard SVM are of high classification accuracy for the broadband and narrowband interferences, but the training speed of LSTWSVM is much faster than the standard SVM. Taking the broadband interference as an example, the training speed of LSTWSVM is 80 times faster than the standard SVM. The monitoring time of the two algorithms are the same (15 μs for both), which satisfies the requirement of the real-time interference monitoring for GNSS signals.

#### 5.2.2. Post-Correlation

For the post-correlation interference monitoring, GPS signals with the broadband and narrowband interferences whose ISRs are 16 dB~24 dB are analyzed. The parameter settings are the same as pre-correlation, and the experimental results are shown in Table 3.

Table 3 shows the comparison of classification accuracy and speed between LSTWSVM and the standard SVM under the broadband and narrowband interferences whose ISRs are both 16–24 dB, and the results indicate that both the LSTWSVM and the standard SVM are of high classification accuracy, but the training speed of LSTWSVM is about 55 times faster than standard SVM. The monitoring time of the two algorithms are the same (both equal to 15 μs), which satisfies the requirement of the real-time interference monitoring for GNSS signals

## 6. Conclusions

Applying SVM in the interference monitoring of GNSS signals satisfies the requirement of objectivity and accuracy. However, the training speed of standard SVM cannot satisfy the requirement of being in real-time for interference monitoring. TWSVM, which is faster than the standard SVM in training speed, is introduced in this paper. The TWSVM model is solved by a modified algorithm, LSTWSVM. LSTWSVM is much faster than the standard SVM, and it can be applied in the field of interference monitoring of GNSS signals. The experimental results indicate that the training speed of TWSVM is on millisecond (ms) level and the classification speed of TWSVM is on microsecond (μs) level, which can be used in practice.

## Figures and Tables

**Table 1:** Broadband interference monitoring result.

Training Algorithm	Interference Strength (dB)	Training Time (ms)	Monitoring Time (μs)	Monitoring Precision (%)
Standard SVM	I/S<5	60	15	100
	5≤I/S<15			100
	15≤I/S<25			100
	I/S≥25			100
LS-TWSVM	I/S<5	0.75	15	100
	5≤I/S<15			100
	15≤I/S<25			100
	I/S≥25			100

**Table 2:** Narrowband interference monitoring result.

Training Algorithm	Interference Strength (dB)	Training Time (ms)	Monitoring Time (μs)	Monitoring Precision (%)
Standard SVM	I/S<5	5.3	15	100
	5≤I/S<15			100
	15≤I/S<25			100
	I/S≥25			100
LS-TWSVM	I/S<5	0.71	15	100
	5≤I/S<15			100
	15≤I/S<25			100
	I/S≥25			100

**Table 3:** Broadband and narrowband interference monitoring results.

Training Algorithm	Interference Strength (dB)	Training Time (ms)	Monitoring Time (μs)	Monitoring Precision (%)
Standard SVM	Broadband I/S:16–24	54	15	100
	Narrowband I/S:16–24			100
LS-TWSVM	Broadband I/S:16–24	0.99	15	100
	Narrowband I/S:16–24			100
